# Study the effect of drying on the oxidation thermogravimetric and functional group composition characteristics of immersed lignite

**DOI:** 10.1038/s41598-022-23375-7

**Published:** 2022-12-14

**Authors:** Wei Guo, Chunhua Zhang, Yongliang Han

**Affiliations:** 1 College of Safety Science and Engineering, Liaoning Technical University, Fuxin, 123000 Liaoning China; 2 China Coal Technology & Engineering Group Xi’an Research Institute, Xi’an, 710077 Shanxi China

**Keywords:** Coal, Chemical engineering

## Abstract

The drying process of immersed lignite has a significant influence on the characteristics and progress of spontaneous combustion. To reveal the influence mechanism of the drying process of immersed coal on the spontaneous combustion characteristics and the change rule of the spontaneous combustion process, in this research, we measured the mass change during coal oxidation with thermogravimetry, and the change of the functional groups with Fourier transform infrared spectroscopy. The influence of the drying process on the coal was analyzed by comparing activation energy, functional group of immersed coal with different drying degrees and raw coal. The results showed that, compared with raw coal, the content of Ar–C–O– and antisymmetric stretching vibration of the carboxylate group (–COO–) as well as the stretching vibration in the quinone group (C=O) and the –OH group increased. For the content of $$\delta s.{\text{RCH}}_{{3}}$$,$$\delta as.{\text{R}}_{{2}} {\text{CH}}_{2}$$$$\delta as.{\text{RCH}}_{{3}}$$, the value of Asym.CH_2_/Asym.CH_3_ decreased. The content of various functional groups changed to be favorable for oxidation and heat release. At different reaction stages, the activation energy was differently affected by the degree of drying. Average values of activation energies at different reaction stages are shown raw coal had the lowest activation energy. After soaking in water and drying, the activation energy of coal is increased to varying degrees, the reactivity is reduced, and the risk of spontaneous combustion is reduced. After soaking in water and drying, the activation energy of coal is increased to varying degrees, the reactivity is reduced, and the risk of spontaneous combustion is reduced. The activation energy of the coal samples dried for 24 h after soaking in water is the lowest among the coal samples dried for different times after soaking in water, and the moisture content is 10.5%.

## Introduction

**Figure 1:**
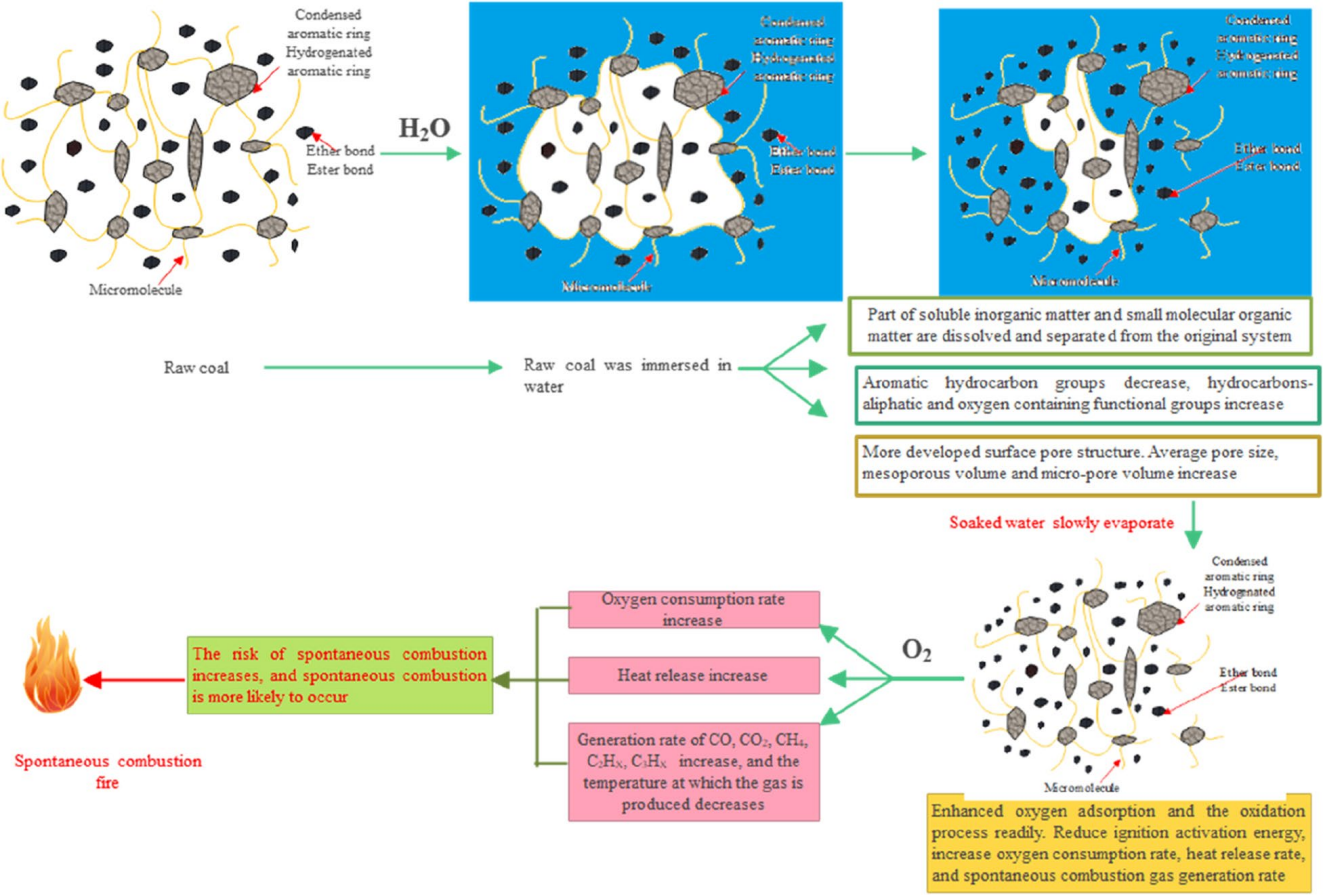
Effect of water immersion and drying on the spontaneous combustion risk of coal.

Coal is a type of porous medium. Water in coal is affected by the van der Waals force and the hydrogen bonds. Only a few functional groups in coal can react with oxygen at low temperatures^1–4^. Qiao et al.^5^ believed that immersed coal had a high active structure, oxidation activity, and heat release; low ignition activation energy; and a strong tendency for spontaneous combustion. With an increase in the water soaking time, the number of side chains of the coal samples increases, and water plays a role in promoting and inhibiting the spontaneous combustion of coal. The dominant role is affected mainly by the soaking time^6^. After the coal is soaked in water, the hydrocarbon aliphatic functional groups increase, the oxygen-containing functional groups increase, the aromatic hydrocarbons decrease, and the oxidation and spontaneous combustion processes of coal accelerate^7–11^. Lu et al.^12^ showed that many active sites accumulated for coal during long-term water leaching. The air-drying of immersed coal is the peroxidation of immersed coal at room temperature. Water immersion can transfer the active sites, but peroxidation can activate the primary active sites^13^. Because of the generation and existence of humid heat, coal may easily undergo oxidation and spontaneous combustion at low temperatures^14^. Wang et al.^15^ pointed out that the presence of water during the PR pyrolysis process showed the competitive characteristics of promoting and inhibiting the spontaneous combustion of the coal at the same time. In addition, the soaking process reduced the crossing point temperature of the soaked coal and increased the risk of spontaneous combustion for the soaked coal^16–18^. After coal is immersed in water, the type, generation rate, and temperature of gas generated during spontaneous combustion change^19–22^. After soaking and air drying, the aliphatic hydrocarbon chain is broken and the chain reaction process is accelerated, which can change the spontaneous combustion oxidation characteristics of coal and promote the oxidation and spontaneous combustion of coal^19,23–25^. These studies have shown that after the coal is soaked in water and air-dried, the hydrocarbon aliphatic group increases, the oxygen-containing functional group increases, and the aromatic hydrocarbon group decreases, which accelerates the oxidation and spontaneous combustion of coal^26–28^. The joint influence mechanism of the changes in the functional group composition and pore structure on the spontaneous combustion of coal during water soaking and air drying is shown in Fig. 1.

The current reaction kinetics cannot fully and accurately explain this phenomenon^29–31^. Therefore, we proposed research to examine the influence of the drying degree of immersed coal. Thermogravity technology was used to measure the change in the quality of coal in the process of spontaneous combustion. An infrared method was used to measure the change of the functional groups in coal. Based on the comparison and analysis of the activation energy, types, and content of functional groups of immersed coal in different air-drying conditions, from the perspective of the changes of activation energy and functional groups of coal samples, the change mechanism of spontaneous combustion risk after coal samples were soaked in water and experienced different degrees of drying in air environment will be revealed.

## Materials and methods

The samples of lignite used in this experiment were obtained from the Fengshuigou Coal Mine of the Pingzhuang Coal Company in Inner Mongolia, China. The moisture content of the raw coal was 10.2%, the ash content was 22.5%, the volatile content of the coal was 42.5%, and the fixed carbon content was 24.8%.

The coal sample was ground up and broken into fragments of less than 0.125 mm, and then the soaking in water for 21 days, Moisture content increased to 20.6%. After soaking in water, the coal samples were taken out, and the excess water was filtered out. Then, the samples were placed in a constant temperature drying oven with an air atmosphere of 35 °C for 8, 24 and 48 h respectively, the moisture content was reduced to 13.1%, 10.5% and 9.4%. After drying in an air atmosphere, the samples were placed in a vacuum drying oven with a vacuum drying temperature of 105 °C for 24 h. The raw coal is not soaked in water and dried under air atmosphere, only dried under vacuum condition at 105 °C for 24 h. The FTIR test is significantly affected by moisture, during the test, the presence of moisture will interfere with the absorption of infrared spectrum. Therefore, it is necessary to process the moisture in the sample before the FTIR test to eliminate the interference of moisture as much as possible. Among them, the vacuum drying can ensure that the coal sample will not cause the change of functional groups due to oxidation. To ensure the consistency between TG-DSC test and FTIR test samples, TG-DSC test samples were processed in the same way.

After drying, a thermogravimetric test and an infrared test were carried out using a synchronous thermal analyzer (STA449F5) produced by Netsch and a Fourier transform infrared spectrometer (FTIR-650) developed by the Tianjin Port East Company, respectively. The heating rates of the synchronous thermal analysis test were 5 K/min, 10 K/min, and 20 K/min, and the airflow was 100 mL/min.

## Experimental results

### Rule of functional group change

**Figure 2:**
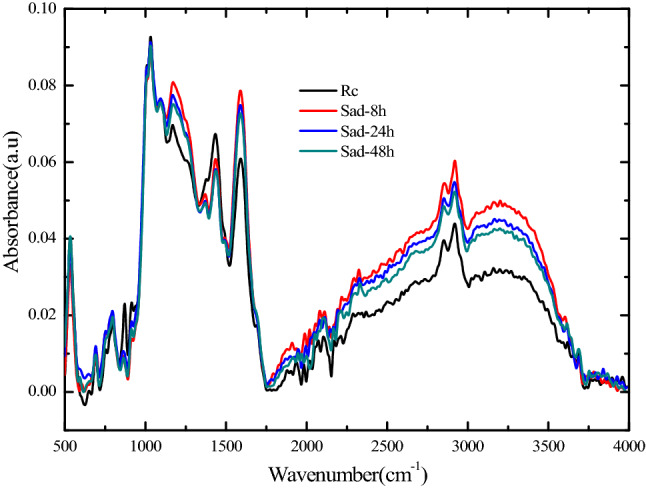
FTIR spectra of coal samples tested in the experiment. Sample label Rc represents the test result of raw coal; Sad-8 h represents the test result after drying for 8 h in the air environment after saturation; Sad-24 h represents the test result after drying for 24 h in the air environment after saturation; and Sad-48 h represents the test result after drying for 48 h in the air environment after saturation.

The surface functional groups were determined with Fourier-transform infrared spectroscopy (FTIR). The FTIR spectra of the coal samples with different treatment methods are shown in Fig. 2. With consideration of the different types of functional groups, the four spectral curves in Fig. 2 were segmented, and the segmented ranges were 1000–1800 cm^−1^, 2800–3000 cm^−1^, and 3000–3600 cm^−1^^32–36^. The fitted curves and peak areas of functional groups were obtained by using PeakFit V4.2 with Gauss-Lorentz function. The confidence coefficients of the fitted curves are higher than 0.99. The total peak area is the sum of individual peak area, and the ratio of the peak area representing certain functional group to the total peak area is the content of the certain functional group in the coal.

**Figure 3:**
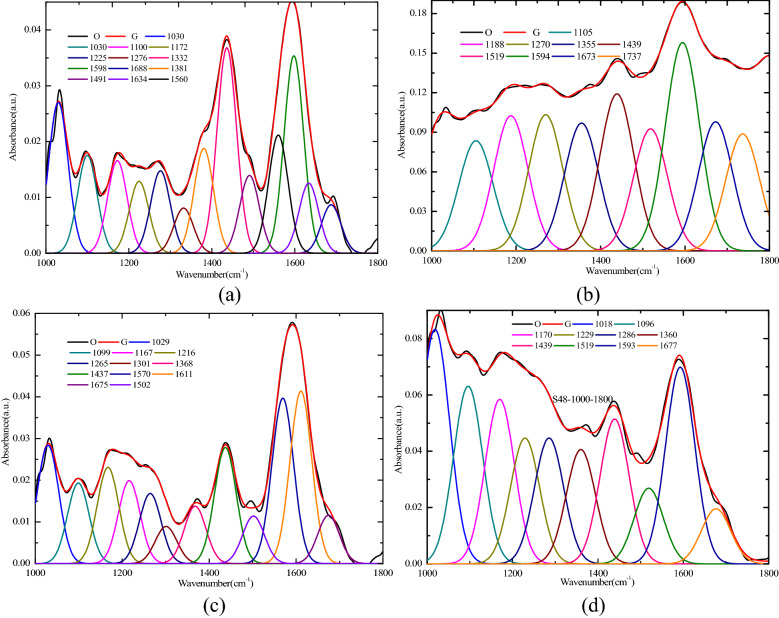
Fitted curve of FTIR spectrum ranging from 1000 to 1800 cm^−1^. ‘O’represents the original test spectral line, and ‘G’represents the newly generated spectral line after multipeak fitting. (**a**) R_C_, (**b**) Sad-8 h, (**c**) Sad-24 h, (**d**) Sad-48 h.

**Figure 4:**
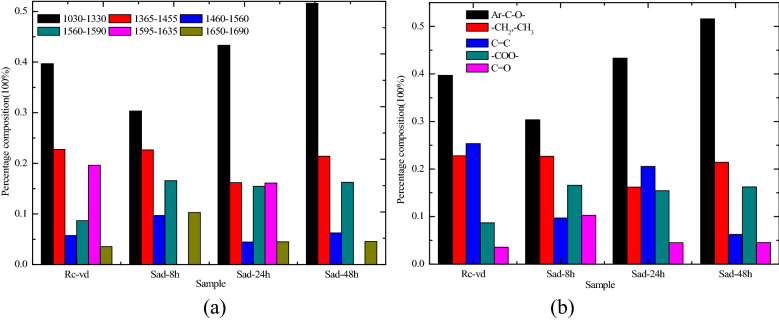
Content distribution of various functional groups for the 1000–1800 cm^−1^ band. (**a**) Percentages of different functional groups (**b**) percentage of different functional groups after the combination of two C=C structures.

The peak fitting results of the 1000–1800 cm^−1^ sections of four coal samples are shown in Fig. 3. The range of 1030–1330 cm^−1^ belonged to the phenol, alcohol, ether, and ester oxygen bond (Ar–C–O–). The range of 1365–1455 cm^−1^ belonged to the scissoring or deformation vibration of the aliphatic structures (–CH_2_, –CH_3_). The range of 1460–1560 cm^−1^ belonged to the aromatic nucleus (C=C). The range of 1560–1590 cm^−1^ belonged to the antisymmetric stretching vibration of the carboxylate group (–COO–). The range of 1595–1635 cm^−1^ belonged to the C=C stretching vibration of the aromatic ring or the condensed ring (C=C). The range of 1650–1690 cm^−1^ belonged to the C=O stretching vibration in the quinone group (C=O)^37–39^. According to the peak area, the percentage content of different functional groups was calculated, as shown in Fig. 4a. For the condition of no oxidation, the C=C in the coal was stable. The two sections of C=C shown in Fig. 4a were combined to draw the content distribution maps of the oxygen-containing functional group in the four coal samples, as shown in Fig. 4b.

The content of Ar–C–O– (phenol, alcohol, ether, and ester oxygen bond) in the three kinds of coal samples with different drying degrees after soaking increased with the drying time, but the content of Sad-8 h was lower than that of Rc, and the content of Ar–C–O– in the Sad-24 h and Sad-48 h was significantly higher than that of Rc. The oxidation activity of the coal samples increased with the increase of the Ar–C–O– content. Compared with the raw coal, the content of scissoring or deformation vibration of the aliphatic structures (–CH_2_ and –CH_3_) in the coal samples at different drying times after soaking in water decreased to different degrees, and the decrease was the largest at Sad-24 h. The decrease in the content of the scisphatic structures suggested that drying after saturation resulted in chain fracture and shortening and increased oxidation activity for the coal. The content of antisymmetric stretching vibration of the carboxylate group (–COO–) and the stretching vibration in the quinone group (C=O) in the drying coal samples was significantly higher than that in the raw coal, which resulted in the oxidation activity of the coal samples after drying being increased^40,41^.

**Figure 5:**
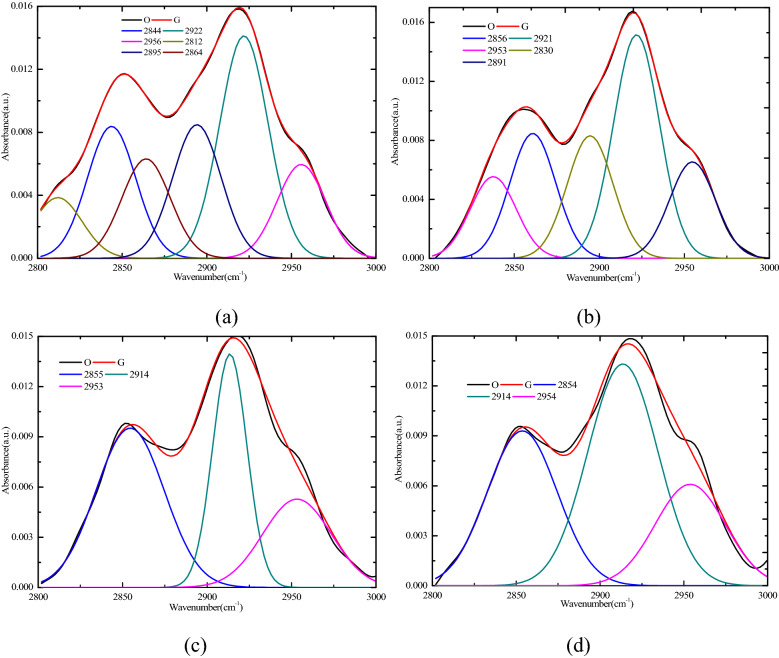
Fitted curve of FTIR spectrum ranging from 2800 to 3000 cm^−1^. (**a**) R_C_, (**b**) Sad-8 h, (**c**) Sad-24 h, (**d**) Sad-48 h.

**Figure 6:**
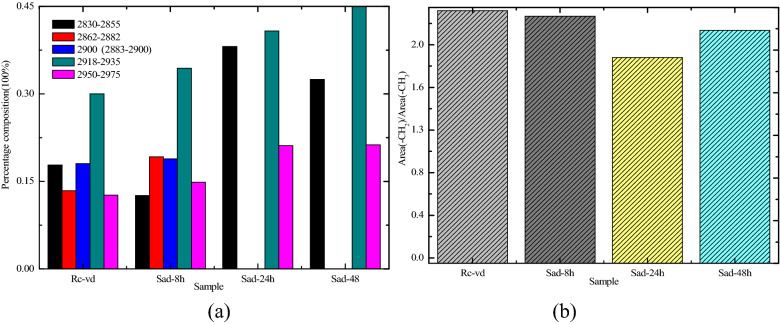
Content distribution of various functional groups for the 2800–3000 cm^−1^ band and asym.CH_2_/asym.CH_3_. (**a**) Percentages of different functional groups (**b**) asym.CH_2_/asym.CH_3_.

The peak fitting results for the 2800–3000 cm^−1^ sections of the four coal samples are shown in Fig. 5. The range of 2830–2855 cm^−1^ belonged to the methylene symmetric stretching vibration (sym.-CH_2_), and the range of 2862–2882 cm^−1^ belonged to the symmetrical stretching vibrations of CH_3_ in the naphthenes or the aliphatic groups (sym.-CH_3_). The value of 2900 cm^−1^ belonged to the –CH in the naphthenes or the aliphatic groups (–CH). The range of 2918–2935 cm^−1^ belonged to the –CH_2_ antisymmetric stretching vibrations of the methyl and methylene groups in the naphthenes or the aliphatic groups as well as to the aromatic methyl groups (asym.-CH_2_). The range of 2950–2975 cm^−1^ belonged to the antisymmetric stretching vibrations of the –CH_3_ in the naphthenes or the aliphatic groups (asym.-CH_3_)^42^. The percentages of the different functional groups are shown in Fig. 6a. The parameter asym.CH_2_/asym.CH_3_, which was applied to estimate the chain length of the aliphatic groups, was calculated according to Eq. (1), as follows:

1 $$ \frac{{{\text{asym}}.{\text{CH}}_{2} }}{{{\text{asym}}.{\text{CH}}_{3} }} = \frac{{A_{2920} \;{\text{cm}}^{ - 1} }}{{A_{2950} \;{\text{cm}}^{ - 1} }}, $$

where A_2920_ and A_2950_ refer to the areas of the fitted subpeaks at 2920 cm^−1^ and 2950 cm^−1^.

The result showed that the value of asym.CH_2_/asym.CH_3_ in different drying degrees for the coal decreased by different degrees, 2.37(Rc), 2.32(Sad-8 h), 1.93(Sad-24 h), and 2.19(Sad-48 h), indicating that the aliphatic chains occurred in different drying degrees of the coal as shorter branches and with more branches (especially in Sad-24 h), as shown in Fig. 6b. Therefore, the molecular structure of the different drying degrees of the coal was less stable due to a loose structure with larger space in the aromatic nucleus.

**Figure 7:**
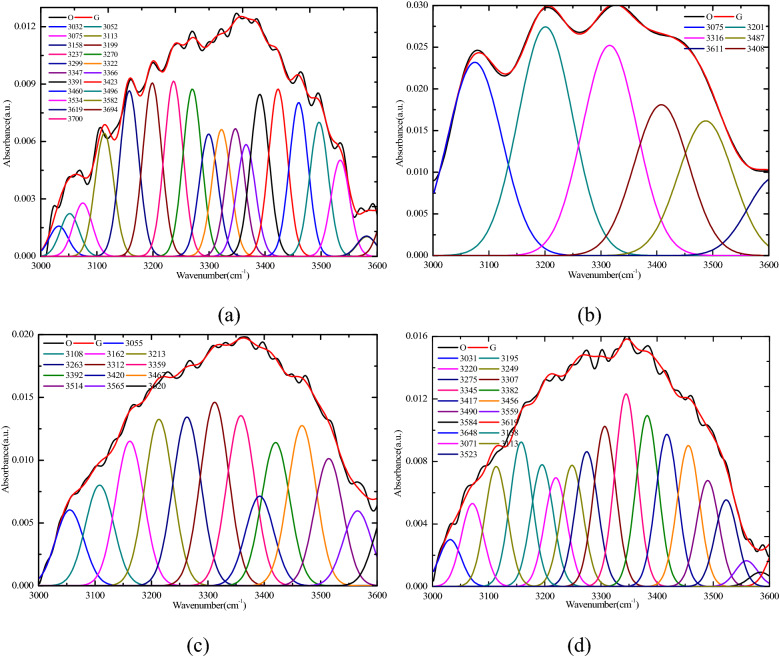
Fitted curve of FTIR spectrum ranging from 3000 to 3600 cm^−1^. (**a**) R_C_, (**b**) Sad-8 h, (**c**) Sad-24 h, (**d**) Sad-48 h.

**Figure 8:**
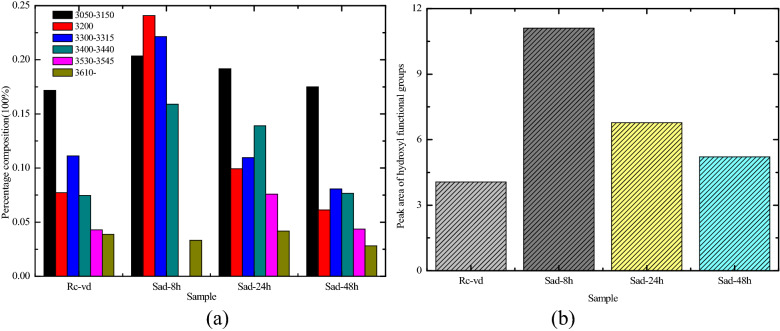
Content distribution of the various functional groups of the 3000–3600 cm^−1^ band and total area of the –OH. (**a**) Percentages of different functional groups (**b**) Total area of –OH.

The fitted curve of the FTIR spectrum ranging from 3000 to 3600 cm^−1^ is shown in Fig. 7. The range of 3050–3150 cm^−1^ belonged to the aromatic C–H stretching, the value of 3200 cm^−1^ belonged to the ring hydrogen bond, the range of 3300–3315 cm^−1^ belonged to the OH⋯OR, the range of 3400–3440 cm^-1^ belonged to the OH⋯OH, the range of 3530–3545 cm^-1^ belonged to OH⋯π, and the value of 3610 cm^–1^ belonged to the free –O H^43^. The content distribution of the various functional groups of the 3000–3600 cm^–1^ band and the total area of the OH were calculated according to the fitting curve in Fig. 7, as shown in Fig. 8. According to Fig. 8a, the content of the ring hydrogen bond (belonging to 3200 cm^–1^), OH⋯OR (belonging to the range of 3300–3315 cm^−1^), OH⋯OH (belonging to the range of 3400–3440 cm^−1^) in the raw coal was lower than that of Sad-8 h and Sad-24 h. The ring hydrogen bond (3200 cm^−1^) and OH (~ 3400 cm^−1^) were prone to be broken because of their low stability and tendency to react preferentially with oxygen^44^. This had a significant effect on the combustion characteristics. The increase of the ring hydrogen bond and the OH led to the risk of spontaneous combustion for the Sad-8 h and Sad-24 h increasing. According to Figs. 7 and 8b, the total area of OH or the content of OH for the raw coal were lower than those for the coal with different drying degrees. Additionally, the hydroxyl functional groups in the molecular structure of the coal were reactive, and a higher proportion of them increased the risk of spontaneous combustion^45^.

Compared with the raw coal, the various functional groups in the different dry coals changed significantly after soaking in water. The content of Ar–C–O– in Sad-24 h and Sad-48 h was significantly higher than that of Rc, the content of scissoring or deformation vibration of the aliphatic structures (–CH_2_ and –CH_3_) in the dry coal samples decreased, and the decrease was the largest at Sad-24 h. The content of the antisymmetric stretching vibration of the carboxylate group (–COO–) and the stretching vibration in the quinone group (C=O) in the drying coal samples was significantly higher than that in the raw coal. All these changes led to an increase in the oxidation activity of the coal samples after drying. The results showed that the value of asym.CH_2_/asym.CH_3_ in the dry coal decreased, indicating that the aliphatic chains occurred in different drying degrees of the coal as shorter branches and with more branches (especially in Sad-24 h). Therefore, the molecular structure of the different drying degrees of the coal was less stable because of a loose structure with larger space in the aromatic nucleus.

The content of the ring hydrogen bond, OH⋯OR, OH⋯OH in the raw coal was lower than that in the Sad-8 h and Sad-24 h. The ring hydrogen bond and the OH were prone to breakage because of their low stability and tendency to react preferentially with oxygen, which had a significant effect on the combustion characteristics. The decrease of the ring hydrogen bond and OH led to the risk of spontaneous combustion for Sad-8 h and Sad-24 h increasing. The total area of OH or the content of OH of the raw coal was lower than that for the coal with different drying degrees, the hydroxyl functional groups in the molecular structure of the coal were reactive, and a higher proportion of them increased the risk of spontaneous combustion.

### Change law of activation energy

**Figure 9:**
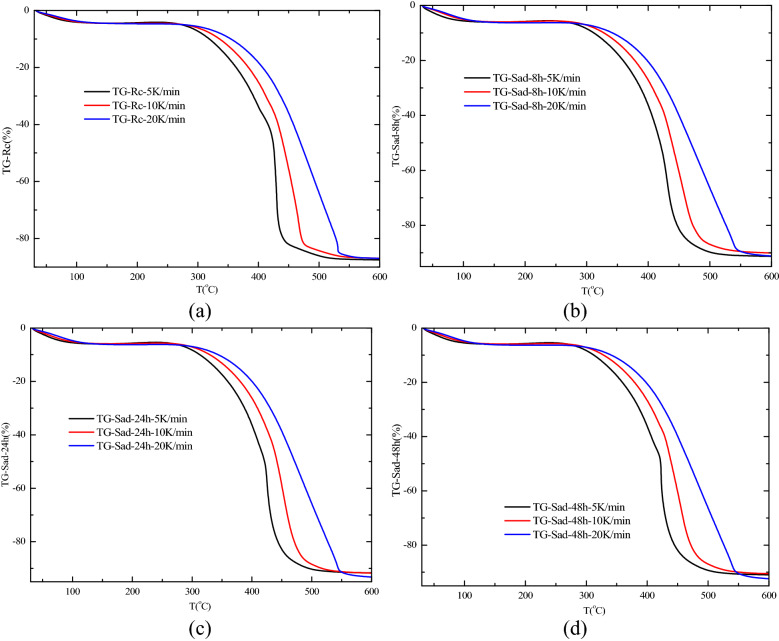
Thermogravimetric curves of coal samples for different heating rates. (**a**) R_C_, (**b**) Sad-8 h, (**c**) Sad-24 h, (**d**) Sad-48 h.

**Figure 10:**
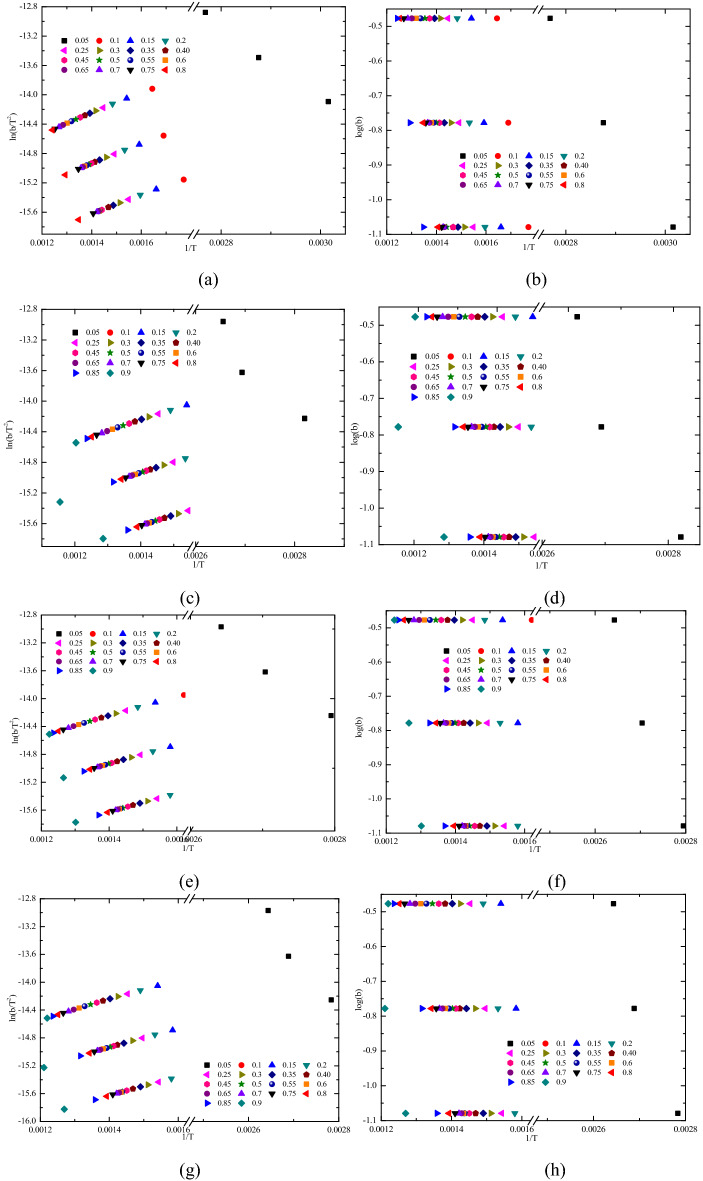
Fitting calculation process of activation energy at different reaction rates. (**a**) R_C_ with the KAS method, (**b**) R_C_ with the OFW method, (**c**) Sad-8 h with the KAS method, (**d**) Sad-8 h with the OFW method, (**e**) Sad-24 h with the KAS method, (**f**) Sad-24 h with the OFW method, (**g**) Sad-48 h with the KAS method, and (**h**) Sad-48 h with the OFW method.

The thermogravimetric curves based on the thermogravimetric (TG) test results for the four groups of samples at heating rates of 5 K/min, 10 K/min, and 20 K/min are shown in Fig. 9. According to the Kissinger–Akahira–Sunose (KAS) and Ozawa–Flynn–Wall (OFW) methods, the temperature corresponding to the reaction rate of 10–90% at three heating rates was taken for the statistics. Generally, when the activation energy of coal is analyzed based on TG data, it is calculated from the reaction rate of 10%. When the reaction rate is lower than 10%, the reaction is unstable due to many external factors of oxidation reaction, which is not suitable for the analysis of activation energy. Therefore, based on the relevant research results, we decided to adopt the activation energy at the stage of reaction rate greater than 10% as the analysis basis. The results are shown in Fig. 10 ^46,47^.

**Figure 11:**
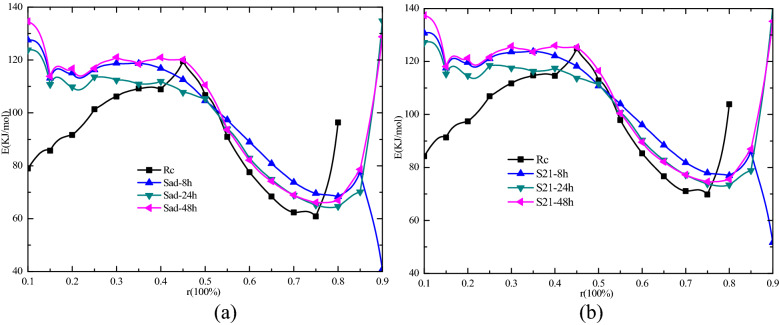
Activation energies (E) corresponding to different reaction rates solved by KAS and OFW methods (r = 0.1–0.9). (**a**) Activation energy (E) for the KAS method, (**b**) Activation energy (E) for the OFW method.

As shown in Fig. 11a, according to the KAS method, the raw coal had the lowest activation energy (E) and the strongest reactivity when the reaction rate was 0.1–0.4. In the range of the reaction rate of 0.45–0.5, the activation energy of the coal sample that was dried for 24 h after soaking in water was the lowest. In other words, among the four experimental samples, the activation energy of the raw coal was the lowest, and the reactivity was the highest, when the response rate is less than 0.4, followed by S21–24 h. The activation energy of the coal samples dried for 24 h were the lowest and the activity is highest, when the response rate is in the range of 0.45–0.5. In contrast, as shown in Fig. 11a and b, the KAS and OFW activation energies obtained in accordance with the relationship between the different coal sample processing for the two noted methods were consistent. For the raw coal, the activation energy of the lowest reactivity was strongest. For 24 h after flooding in the air environment for the dry coal samples, the activation energy was the lowest in different degrees of dry coal samples, and the activity was the strongest. According to the activation energy results solved with the TG curves at different heating rates within the response rate range of 0.1–0.45, the spontaneous combustion risk of the raw coal in this experiment was the highest, followed by the coal samples dried for 24 h after soaking in water, followed by the coal samples dried for 8 h after soaking in water, and the lowest risk was that for the coal samples dried for 48 h after soaking in water.

In the reaction rate range of 0.5–0.75, the lowest activation energy was that for raw coal, and the activation energies for the coal samples dried for 48 h and 24 h were slightly higher than that of raw coal; the two were the same. The highest activation energy was that for the coal samples dried for 8 h after soaking in water, indicating that the raw coal had the highest oxidation reaction activity with oxygen at that stage, followed by the coal samples dried for 24 h and 48 h after soaking in water. When the reaction rate reaches 0.75–0.8, the activation energy of raw coal rises abruptly, while other coal samples begin to increase significantly after the reaction rate of 0.8. When the reaction rate of 0.8, the activation energy of raw coal is the highest.

In the process of TG test, the reaction rate of raw coal did not reach 90%, while the reaction rate of other dried coal samples after immersion in water all reached above 90%, which was also the consequence brought by immersion and drying after immersion. According to the relevant determination methods of activation energy, the average activation energy at the stage from 10% to the maximum reaction rate should be selected as the evaluation standard. Therefore, we averaged the activation energy at the stage from 10 to 80% calculated by KAS method, and the average activation energy of Rc, Sad-8 h, Sad-24 h, and Sad-48 h, is 91, 101.5, 97.1, 101.7 kJ/mol. The activation energy of 10%-80% stage calculated by OFW method is averaged, and the average activation energy of raw coal is 97.5, 107.5, 103.4, 107.7 kJ/mol.

According to the average value of activation energy solved by KAS and OFW, the calculation results of the two methods are close, and the relative error is between 5.9 and 7.2%, indicating that both methods can be used to solve the activation energy, and both methods show that raw coal has the lowest activation energy and the highest activity. The second is Sad-24 h, and Sad-8 h and Sad-48 h are basically the same, which also proves that the activation energy of coal samples soaked in water and dried in the air environment increases, and the reactivity decreases.

## Conclusion

1. Compared with raw coal, the content of phenol, alcohol, ether, ester oxygen bonds (Ar–C–O–), and antisymmetric stretching vibration for the carboxylate group (–COO–), as well as stretching vibration in the quinone group (C=O), and OH⋯OR, OH⋯OH, OH⋯π, and free –OH in the saturated coal increased after drying for different times. For the content of scissoring or deformation vibration of the aliphatic structures ($$\delta s.{\text{RCH}}_{{3}}$$,$$\delta as.{\text{R}}_{{2}} {\text{CH}}_{2}$$$$\delta as.{\text{RCH}}_{{3}}$$), the value of Asym.CH_2_/Asym.CH_3_ decreased. The content of various functional groups was favorable for oxidation and heat release, and changes in the coal samples dried for 24 h and dried for 8 h were the most significant.
2. The results for the coal thermogravimetric experiment and the thermodynamic calculation showed that the activation energies calculated with the KAS and OFW methods were basically consistent within the range of 10–80% after the coal reacted with oxygen at the heating rate of 5–20 K/min. According to the change rule of the activation energy, the raw coal had the lowest activation energy, the strongest reactive activation, and the highest risk of spontaneous combustion. After the raw coal is immersed in water, the reaction activity of air-drying decreases and the risk of spontaneous combustion decreases.
3. Compared with raw coal, the changes of activation energy and functional groups of coal samples after soaking in water and drying for different times have certain differences in explaining the reactivity and spontaneous combustion risk of coal samples, and sometimes even opposite. Therefore, it is not possible to judge the change result of spontaneous combustion risk of coal samples by relying on one or several changes alone. The change of some functional groups after soaking in water and drying may lead to the increase of spontaneous combustion risk of coal, while the result of activation energy shows that the reactivity of dried coal samples after soaking in water decreases and the risk of spontaneous combustion increases, which also includes an important possible reason, that is, the effect of soaking and drying process on the pore structure of coal. Therefore, In the following research, to accurately explain the change law of reactivity and spontaneous combustion risk of coal after immersion and drying, it is necessary to carry out the test of the change law of pore structure and the experimental study of coal spontaneous combustion process under the condition of multiple changes of external factors.

## Data Availability

The data used to support the findings of this study are included within the article.
